# Supplementary figures and images for: Adaptation to life on land at high O2 via transition from ferredoxin-to NADH-dependent redox balance
Source: Proc Biol Sci. 2019 Aug 21;286(1909):20191491. doi: 10.1098/rspb.2019.1491 (PMC6732389; doi:10.1098/rspb.2019.1491)

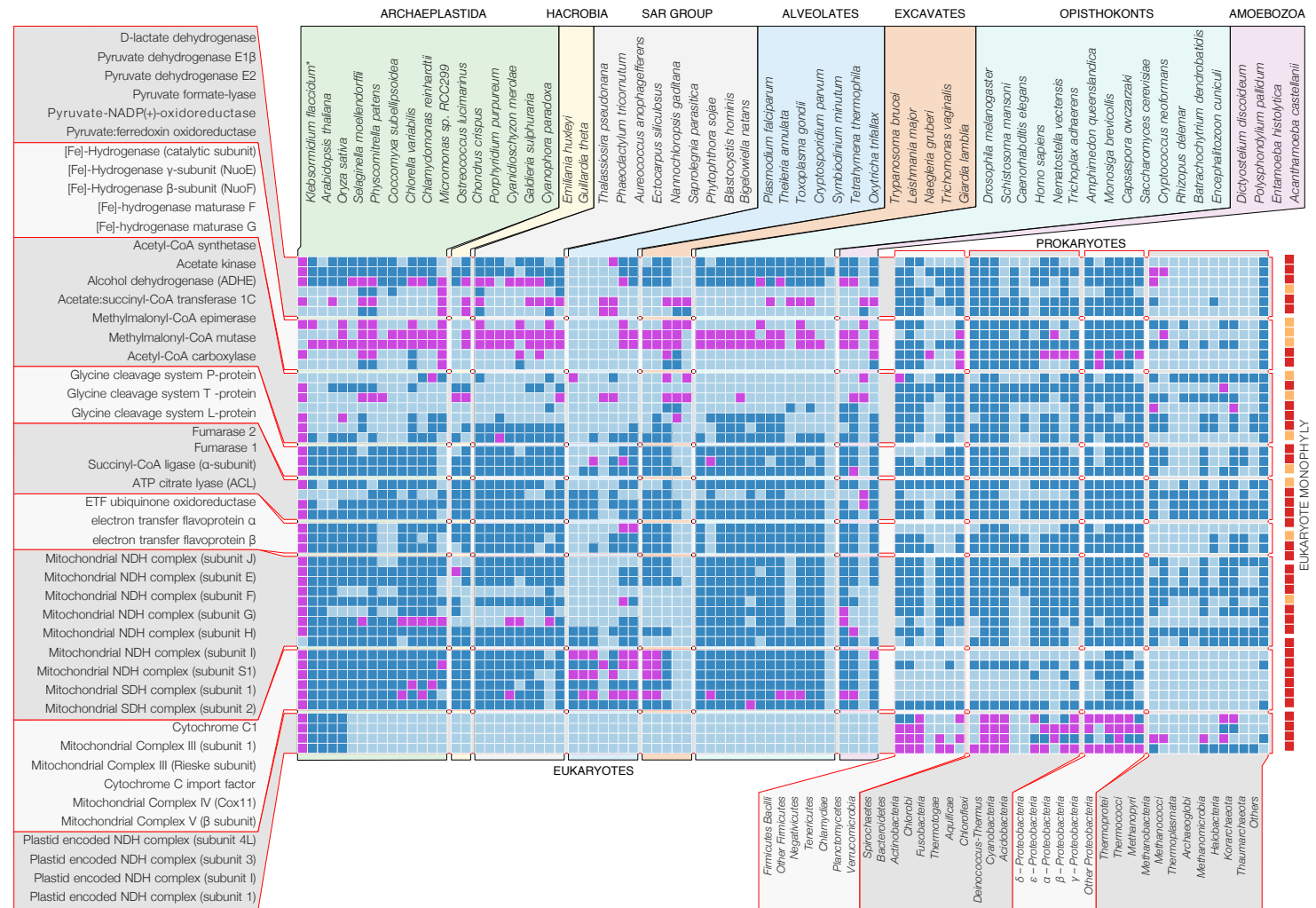

Supplement: Figure S1 [file rspb20191491supp1.pdf]

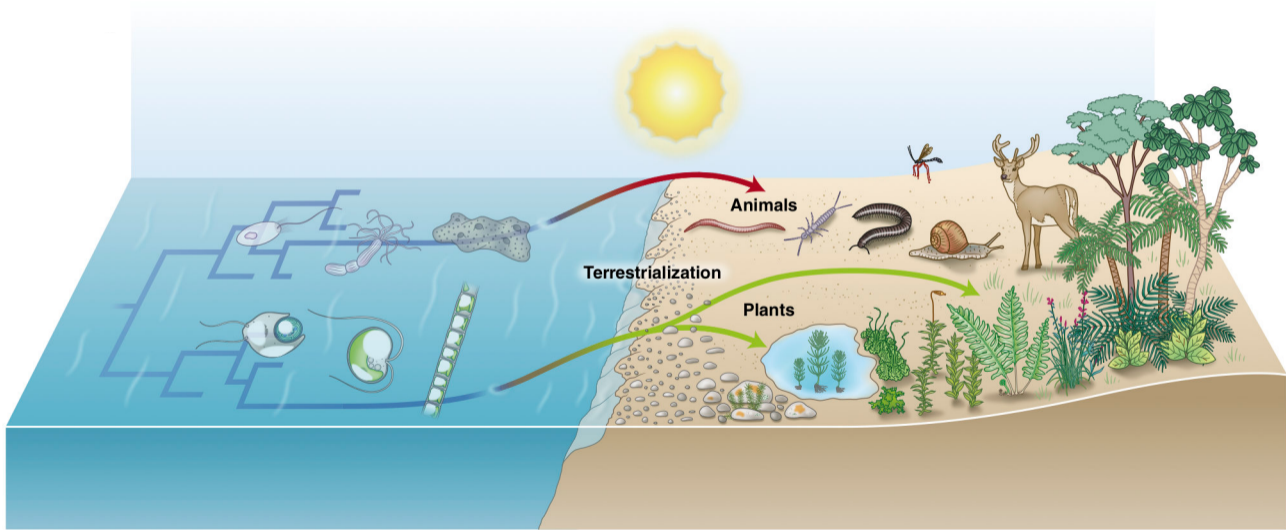

Supplement: Figure S2 [file rspb20191491supp2.pdf]
